# Pyrolysis of Waste Tires: A Review

**DOI:** 10.3390/polym15071604

**Published:** 2023-03-23

**Authors:** Wenwen Han, Deshang Han, Hongbo Chen

**Affiliations:** 1 National Engineering Research Center of Advanced Tire Equipment and Key Materials, Qingdao University of Science & Technology, Qingdao 266061, China; hbhanwenwen@qust.edu.cn; 2 Shandong Key Laboratory of Advanced Manufacturing of Polymer Materials, Qingdao 266061, China; 3 College of Electromechanical Engineering, Qingdao University of Science & Technology, Qingdao 266061, China

**Keywords:** waste tires, pyrolysis, temperature, catalysts, products

## Abstract

Waste tires are known as “black pollution”, which is difficult to degrade. The safe handling and recycling of waste tires have always been the focus of and difficulty for the global rubber industry. Pyrolysis can not only solve the problem of environmental pollution but also completely treat the waste tires and recover valuable pyrolysis products. This paper summarizes research progress on the pyrolysis of waste tires, including the pyrolysis mechanism; the important factors affecting the pyrolysis of waste tires (pyrolysis temperature and catalysts); and the composition, properties, and applications of the three kinds of pyrolysis products. The composition and yield of pyrolysis products can be regulated by pyrolysis temperature and catalysts, and pyrolysis products can be well used in many industrial occasions after different forms of post-treatment.

## 1. Introduction

Tires are made of natural rubber (NR) and synthetic rubber (SR). NR is an important strategic resource and is composed of an elastic polymer from latex (cis-1,4-polyisoprene). NR is a variety of polymer compounds and is composed of different monomers under the action of the trigger agent, and there are different types of monomers, such as butadiene, styrene, propylene, isobutene, neoprene, and so on [1,2]. Tires are prepared through NR and SR being mixed and then vulcanized. After vulcanization, tires themselves have three-dimensional cross-linked chemical structures, which makes it difficult for the tires to biodegrade and photochemically decompose under natural conditions [3]. Tires have a high carbon content and are a type of high-calorific-value fuel (the calorific value is about 35 MJ/kg, which is equivalent to the calorific value of coal). Tires made of rubber and various rubber products have been widely used throughout the world, resulting in a large amount of hard-to-decompose waste rubber. Unreasonable handling of this material will cause economic and environmental problems.

**Figure 1:**
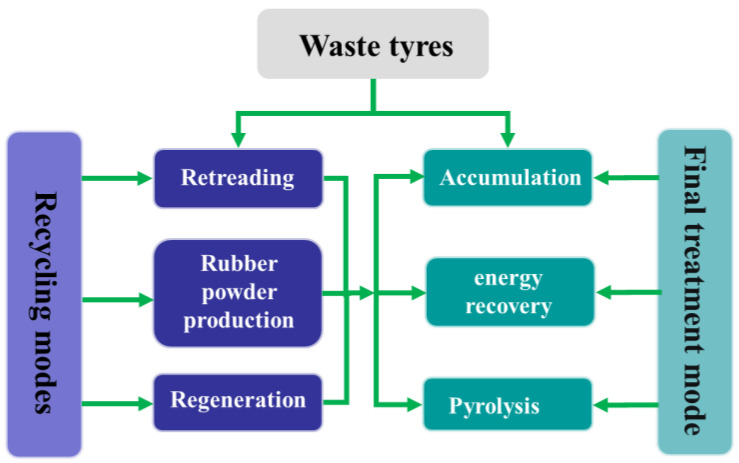
Main methods of WT treatment.

The main methods of waste tire (WT) treatment are tire retreading, rubber powder production, heat energy utilization, pyrolysis, and so on, as shown in Figure 1. Rubber products can no longer be used to produce rubber products after being recycled two to three times, so they must be disposed of eventually. The production cost of rubber powder is high, and the demand is limited. The production process of regenerated rubber is complicated, and the waste gas produced will do great harm to the environment if not treated properly. The accumulation of WT not only occupies land resources but also easily breeds mosquitoes, which spread diseases. Although the heat energy utilization of WT can be utilized with its high calorific value properties, it will cause secondary pollution to the environment. Pyrolysis can decompose WT completely, and its products such as oil, gas, carbon black and steel wire can be used, which not only realizes the regeneration of resources but also solves the problem of pollution. Therefore, pyrolysis technology is an important method and direction of WT recycling at present.

Pyrolysis consists of heating the WTs to a certain temperature so that the pyrolysis reaction occurs; the large molecular chains are broken into small molecular chains, and finally, three types of pyrolysis products are obtained, namely pyrolysis oil, pyrolysis gas, and pyrolysis carbon black [4]. Pyrolysis can thoroughly dispose of a large amount of waste rubber and at the same time obtain high-value pyrolysis products, such as oil, chemicals, carbon black, and fuel gas with high calorific value, which is considered a more promising treatment method, not only protecting the environment but also increasing economic benefits [5].

Due to the obvious advantages of pyrolysis in WT treatment, there are many related studies, and some related reviews have been published. The published reviews on WT pyrolysis have focused on some aspects, such as pyrolysis reactors [6,7,8], mathematical models of pyrolysis process [9], modification of pyrolysis carbon black [10], catalysts applied during pyrolysis [7], and so on. The key to the industrialization of WT pyrolysis is to obtain high-quality pyrolysis products while minimizing energy consumption in the pyrolysis process. This review focuses on the important factors affecting the pyrolysis of WTs (pyrolysis temperature and catalysts) and the composition, properties, and applications of the three types of pyrolysis products. The composition and yield of pyrolysis products can be regulated by pyrolysis temperature and catalysts, and pyrolysis products can be well used in many industrial applications after different forms of post-treatment.

### 1.1. Sources of Large Quantities of WTs

**Figure 2:**
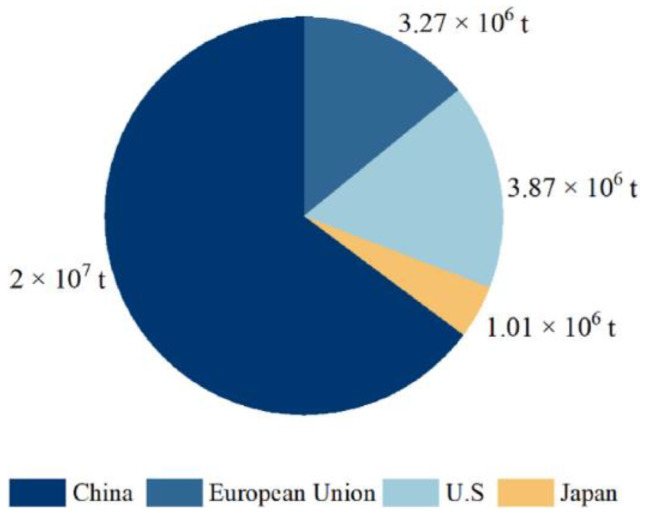
The annual production of WTs in some countries [13] (adapted with permission from Elsevier).

The polymer material used in making tires is mainly composed of NR and SR. Due to the special high elasticity, excellent wear resistance, shock absorption, insulation, and sealing performance, rubber products are widely used in industry and our lives, bringing great convenience to people’s lives. Today, the surge in the number of cars and the booming rubber industry has led to a growing global demand for rubber. Approximately 1.5 billion tires are sold worldwide each year, of which 50 percent are discarded without any treatment [11]. It is widely believed that every new tire sold on the market will have another tire scrapped. The annual production of WTs in some countries is depicted in Figure 2. It is estimated that by 2030, 1200 million WTs will be produced each year [12]. Therefore, both now and in the future, the world is faced with the worldwide problem that it is difficult to recycle the large number of WTs produced each year.

### 1.2. Composition of Tires

Tires are mainly composed of rubber, carbon black, and a variety of organic and inorganic additives (including plasticizers, anti-aging agents, sulfur and zinc oxide, etc.) [14]. There is NR (20–25%), styrene butadiene rubber (SBR) (30–50%), butyl rubber (BR) (up to 30%), carbon black (30%), sulfur (1–2.5%), and a small amount of organic and inorganic additives in the general tread rubber on the market. The proportions of ingredients in the formula vary mainly depending on the purposes of use [9,15]. The SR used in tread compound formula is mainly SBR and BR. In the production process of tires, NR and SR were mixed in a certain proportion and cross-linked through sulfur reaction to form a very stable three-dimensional cross-linked chemical structure, which plays a bearing, damping, and anti-wear role.

### 1.3. Disposal of WTs

At present, the main treatment methods of WTs are direct use, landfill, direct incineration, old tire refurbishment, reclaim, and pyrolysis [12,16,17]. Vulcanized rubber consists of long-chain polymers (isoprene, butadiene, and styrene), which are cross-linked with sulfur bonds and are further protected by antioxidants and anti-ozone agents [18]. Improper treatment can cause great pollution of the environment. Landfilling or directly discarding will contaminate land and water sources as the rubber can take centuries to degrade [19], and the accumulation of tires will provide mosquitoes with breeding grounds, spread disease, and become fire hazard sources, causing serious pollution to the environment and threatening human survival [20]. Direct incineration will release dioxins, polycyclic aromatic hydrocarbons, and volatile toxic pollutants [21,22]. Large amounts of harmful gases will pollute the atmosphere, and waste residues and some heavy metals will seriously pollute soil and water resources [23]. Direct use and tire refurbishment are our preferred resource-saving methods, and direct use can be used in pendants, playgrounds, shoes, etc., but the consumption is small and not enough to deal with the WTs produced in large quantities every year. Tire refurbishment requires the tire body to remain intact and is limited by the number of renovations. There are some problems in the preparation of the reclaimed rubber, such as low profit, high energy consumption, and serious pollution. At the same time, reclaimed rubber only realizes the reuse of resources; the product will eventually become waste rubber [24].

At present, pyrolysis is considered to be the most efficient and thorough method of treating WTs; it can not only recover high-value pyrolysis products and realize the regeneration of resources but also solve the problem of environmental pollution [25,26,27]. Therefore, the pyrolysis technology is an important method for handling waste rubber.

## 2. The Pyrolysis Mechanism of WTs

**Figure 3:**
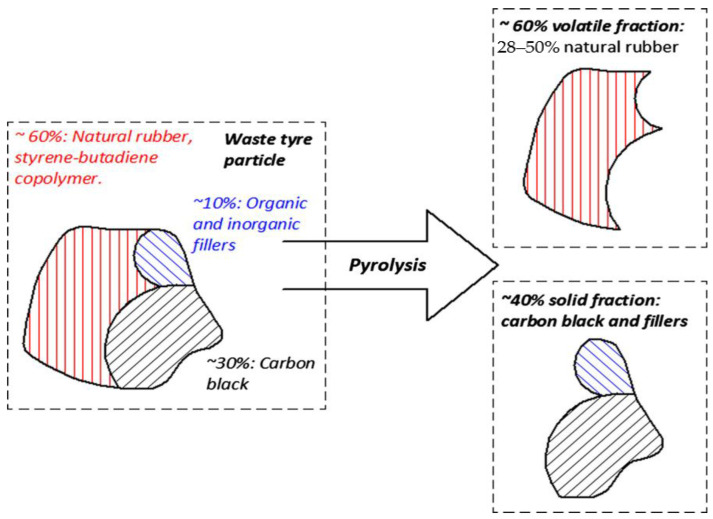
The scheme of WT pyrolysis [6] (Adapted with permission from Elsevier).

**Figure 4:**
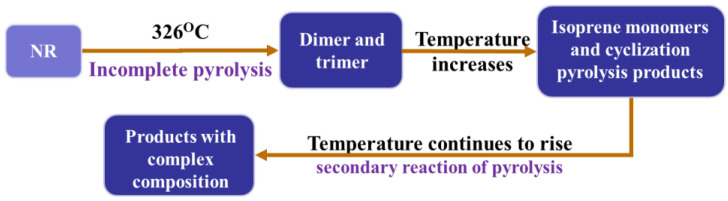
The pyrolysis process of NR.

WT consists of 60% NR and SR, 30% carbon black, and 10% organic and inorganic fillers. After pyrolysis, waste tires are decomposed into 60% volatile fraction and 40% solid fraction, as shown in Figure 3. In other words, WT pyrolysis mainly includes the pyrolysis of two organic components, namely NR and SR. For NR, when the pyrolysis temperature reaches 326 °C, the incomplete pyrolysis reaction begins. At this time, the pyrolysis products are mainly dimer and trimer. As the temperature increases, a large number of isoprene monomers are produced, and cyclization pyrolysis products—such as xylene—are generated at the same time. As the temperature continues to rise, the secondary reaction of pyrolysis products will happen and the product composition will become more complex [28]. The whole pyrolysis process of NR is shown in Figure 4.

**Figure 5:**
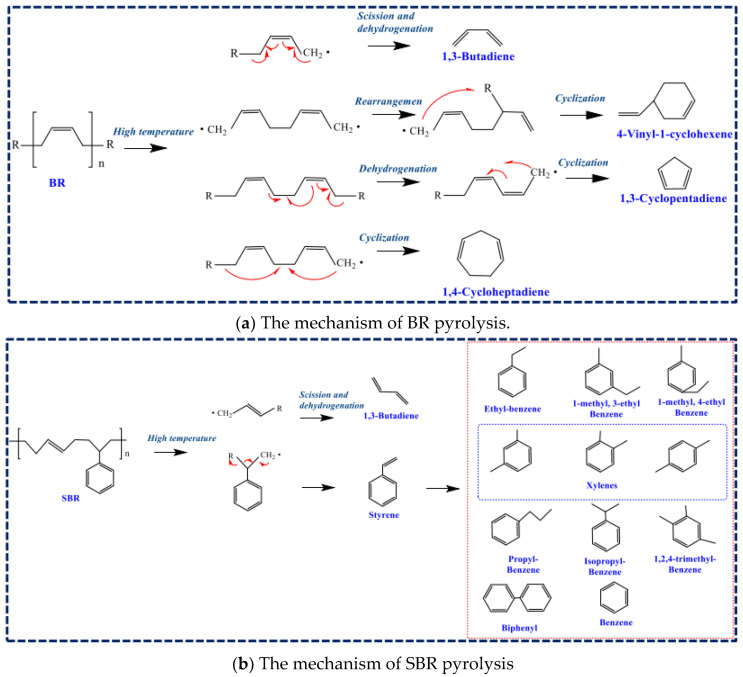
The mechanism of SR pyrolysis; (**a**) BR, (**b**) SBR [29] (adapted with permission from Elsevier).

SR in WTs is mainly composed of butadiene rubber (BR) and styrene–butadiene rubber (SBR). The pyrolysis mechanism of BR and SBR was proposed based on TG-FTIR/MS and Py-GC-TOF/MS [29]. For BR, there were four paths for compound transformation. Once heated, free radicals would appear. The first path was the formation of 1,3-butadienes via scission and dehydrogenation. The second method encompassed the rearrangement and cyclization of free radicals, ultimately triggering 4-vinyl-1-cyclohexenes. The other two methods occurred at almost the same time. One was the formation of 1,3-cyclopentadienes via dehydrogenation and cyclization. The other was the process of cyclization, forming 1,4-cycloheptadienes. The procedure for BR thermal cracking is shown in Figure 5a. For SBR, the process of pyrolysis covered a wide temperature range from 180 °C to 500 °C. Free radicals with C4 mainly went to 1,3-butadienes. Other free radicals of benzene derivatives mainly turned into styrene. Then, reaction between benzene derivatives occurred. The transformation path of SBR under hyperthermic and anoxic conditions is shown in Figure 5b.

In the process of WT pyrolysis, there are two main reactions: main chain degradation and cross-bonding disconnection to form active chain segments. First of all, the broken chain occurs in C-C bonds, and the fracture is accompanied by hydrogen transfer, resulting in a decrease in the molecular weight of the chain segment [30]. Second, active bond fragments containing sulfur radicals may recombine to form a new network. It can be seen from mass spectrometry (MS) analysis of rubber pyrolysis, the pyrolysis products break from the main chain to form monomer or dimer of polymer, which indicates that the dissociation energy of C-C bonds is lower than that of C-H bonds. When heated, C-C bonds are prone to fracture, and free radicals are formed. Then, the free radicals react with each other to extract hydrogen or conduct disproportionation reactions to form a variety of products. The results of magnetic resonance imaging (MRI) showed that the fracture of single sulfur bonds mainly occurred at 300 °C, and with the extension of pyrolysis time, the isoprene unit in NR changed from cis to trans isomerization. At the range from 280 °C to 300 °C, the cross-linked bond fracture of NR occurred most frequently, followed by a rapid increase in the degradation of the main chain, and the higher the temperature, the higher the efficiency of this process. This indicated that the cross-linking fracture occurred earlier in the rubber pyrolysis, mainly because the dissociation energy of S-S bonds is lower than that of C-S bonds. However, with the extension of pyrolysis time, the main chain fracture became more obvious [31]. The pyrolysis of NR is dominated by the fracture of main chain and crosslinked bonds, with a low probability of recombination in the chain fracture [32].

**Figure 6:**
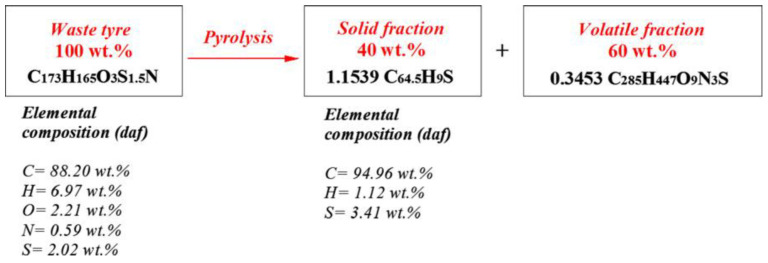
Elemental analysis of tire pyrolysis process (*daf* means dry ash-free basis) [6] (adapted with permission from Elsevier).

In short, the pyrolysis reaction is a very complex process which cannot be completely described by one or several chemical reactions. However, the process is based on the mass conservation law, and the empirical formula with a guiding value can still be obtained. In the process of pyrolysis, WTs were decomposed into solid carbon black and volatile products. Martínez et al. [6] assumed that the solid conversion rate is 40% and that there was only interaction between organic components in the pyrolysis reaction. Based on this assumption, the pyrolysis process was expressed in the form of elemental analysis (as shown in Figure 6). On the basis of element analysis, the enthalpy of reaction of tire pyrolysis can be added to obtain the energy content of volatilization through heat balance.

In addition, the formation mechanism of the pyrolysis products was also studied. Xu et al. [33] conducted pyrolysis on waste bicycle tires and found that the pyrolysis process could be divided into two stages. In stage I, the primary pyrolysis of tires occurred at 285–531 °C, while in stage II, the secondary pyrolysis of pyrolysis products occurred mainly at 663–847 °C. According to thermogravimetric analyzer coupled with Fourier-transform infrared spectrometry (TG-FTIR), in stage I, small molecule alkenes and cycloalkenes were produced when rubber chains were broken at low temperatures. With the increase in pyrolysis temperature, the alkenes and cycloalkenes produced underwent Diels–Alder reaction, cyclization, aromatization and other reactions to form benzene and benzene derivatives. At the same time, more alkenes and cycloalkenes were ring-opened and recycled. In stage II, as the temperature rose again, benzene and its derivatives underwent further pyrolysis to obtain more aromatic compounds. Wei et al. [32] combined reactive molecular dynamics (RMD) simulation and TG-FTIR experiment to study the formation process of NR pyrolysis gas. The simulated results showed that CH_3_ was separated from the main chain and that H was extracted from other molecules to generate CH_4_, while C_2_H_4_ was generated mainly through C-C bond fracture from the long chain. Other small gas molecules were produced by breaking down large alkenes or low-activity free radicals. Seidelt et al. [34] also detected the thermal decomposition of SBR, NR, and BR by gas chromatographic/mass spectrometric (GC/MS). The results showed that the main pyrolysis products of NR were xylene and isoprene, while the main pyrolysis products of SBR were ethylbenzene, styrene, and cumene. Ding et al. [35] found that chain olefins were mainly derived from 2-pentene, 1-3-butadiene, and isoprene formed through the depolymerization of NR. Lopez et al. [36] found that cyclic olefin was mainly derived from the degradation of NR or the cyclization of chain olefin. The degradation process and secondary reactions mainly included terminal chain breaking, random chain breaking and cross-linking. The degradation process was studied via thermogravimetric analysis (TGA), and the results showed that with the increase in temperature and heating rate, the chain breaking rate of cross-linking decreased. Studies have shown that the activation energy of rubber polymer decreased with the increase in heating rate (171.06–136.51 kJ/mol) [37]. Under the condition of low pyrolysis temperature, chain uncoupling and chain breaking played a leading role. With the increase in pyrolysis temperature and activation of coupling reaction, the cross-linking mechanism played a more effective role in the pyrolysis process, and the products were transformed into cyclic compounds, which was consistent with other research results [38].

Pyrolysis is a complex decomposition process of organic matter, including both chemical and physical reactions [39]. In the process of pyrolysis, different organic compounds start to decompose at different temperatures, and the reaction and product composition are also different at different pyrolysis temperature. The pyrolysis of macromolecular organic matter includes macromolecular bond breaking, molecular isomerization and small molecular organic polymerization. The thermal stability of polymer mainly depends on the formation of bonds and the bond energy between atoms. The greater the bond energy, the harder it is to break and the higher its thermal stability. The smaller the bond energy, the easier it is to decompose and the lower its thermal stability. The composition and yield of the pyrolysis products depend on the raw material, pyrolysis temperature, pyrolysis rate, catalyst, and so on. The revelation of pyrolysis mechanism of NR and SR can not only help us understand the pyrolysis process but also effectively guide the regulation of pyrolysis products.

## 3. The Important Influencing Factors of WT Pyrolysis Process

WT pyrolysis is the process in which the rubber polymers that make up WTs are decomposed into gas, oil and carbon black at the right temperature in the absence of oxygen or presence of inert gas. There are many factors that influence the pyrolysis process, such as reactor structure, raw material composition, pyrolysis temperature, pyrolysis pressure, pyrolysis residence time, heating rate, catalyst, waste rubber particle size, etc. Among them, pyrolysis temperature and catalyst are important factors influencing the pyrolysis reaction [40,41,42,43,44,45]. The influences of these two factors on the pyrolysis process and pyrolysis products of WTs are reviewed as below.

### 3.1. Temperature

The long rubber polymer chains can only undergo condensation, polymerization, depolymerization, hydrogenation, and aromatization reactions at appropriate temperatures, and an important factor influencing this series of reactions is temperature [18]. In the following section, the pyrolysis process of rubber at different temperatures and the influence of temperature on the pyrolysis products are summarized.

#### 3.1.1. Pyrolysis Process of WTs at Different Temperatures

At present, researchers mainly use thermogravimetric (TGA) and MS to analyze the pyrolysis characteristics of WTs. Tires are usually made of NR and SR. According to the results of TGA, the initial temperature of the pyrolysis reaction of NR was 326 °C, the pyrolysis speed reached its maximum at 375 °C, and the pyrolysis process was completely finished when the temperature reached 455 °C; when the pyrolysis temperature reached 286 °C, the pyrolysis reaction of SBR started. The pyrolysis speed reached its maximum at 452 °C, and the pyrolysis process was completely finished when the temperature reached 491 °C. The initial pyrolysis temperature of BR was 374 °C, the pyrolysis speed reached the maximum at 483 °C, and when the pyrolysis temperature reached 497 °C, the pyrolysis process was basically completed [46]. According to the above results, NR is decomposed first, followed by SBR and BR over the entire pyrolysis process.

Han et al. [47] divided the WT pyrolysis process into four stages according to MS and TG curves. In the first stage, the temperature was lower than 320 °C, the water in the tire was evaporated, and the plasticizer was decomposed; the second stage is at the temperatures between 320 °C and 400 °C, when the decomposition of NR occurred; the third stage occurred at 400–520 °C, and the decomposition of SBR and BR took place; the fourth stage occurred when the temperature was above 520 °C, with a small reduction in mass. Meanwhile, Kan et al. [48] found through experiments that there were three stages of WT pyrolysis: the first stage (about 200–350 °C) was the decomposition of volatile substances (such as oil, plasticizer, additive, etc.) in rubber, the second stage was the decomposition of NR component at about 300–450 °C, and the third stage was the degradation of SBR and BR components at about 400–500 °C. Islam et al. [49] believed that the decomposition temperatures of organic additives, such as oil and plasticizer, in tires were about 150–350 °C, 330–400 °C for NR, and 400–480 °C for SBR and BR. It can be seen from the above that the pyrolysis sequence of WTs can be divided into four main stages successively: at about 200 °C, due to the decomposition of additives at low boiling point, waste tires began to lose weight; NR began to decompose at about 300 °C; SR did not begin to decompose until 400 °C; pyrolysis was basically completed at about 500 °C.

#### 3.1.2. Effect of Temperature on Pyrolysis Products

Different types of tires, different experimental conditions, experimental equipment, experimental operations, and different catalysts may lead to different or even opposite experimental results. Even if the same type of waste rubber is pyrolyzed at the same temperature, different experimental results will be obtained. Most of the researchers found that with the increase in pyrolysis temperature, pyrolysis oil production of WTs decreased and gas production increased [36,50,51]. Other researchers found that pyrolysis oil production would reach the maximum or minimum at a certain temperature [52,53]. The former had a high degree of recognition, but there was also a group of the researchers who found that the production of pyrolysis oil increases with the increase in pyrolysis temperature [54]. In a word, temperature has a great influence on the oil production of waste tire pyrolysis. Pyrolysis oil production at different temperatures is shown in Table 1.

When the WTs are pyrolyzed at different temperatures, the main pyrolysis products also change. When the temperature is low, the secondary reaction of macromolecular organic compounds can be reduced and more molecular chains above C_5_ can be generated, so the pyrolysis oil content will be higher. When the pyrolysis temperature is higher, the large rubber molecular chain will have a large-scale stable bond breaking reaction without enough time to decompose at the weakest molecular chain node, and the small molecular chain generated by pyrolysis will have a secondary reaction. Therefore, low molecular organic compounds are mainly generated, resulting in an increase in olefin gases and a decrease in oil content. In general, increasing pyrolysis temperature will increase gas production and decrease oil production. The higher the pyrolysis temperature is, the faster the secondary pyrolysis reactions will be—such as cyclization, dehydrogenation, and aromatization—which will reduce the aliphatic components and increase the aromatic components in the pyrolysis oil [52]. Berrueco et al. [59] conducted pyrolysis of WTs at 400–700 °C. When the pyrolysis temperature varied from 400 °C to 500 °C, oil production increased, but when the pyrolysis temperature reached above 500 °C, the oil production did not increase any more. Gas production increased slightly at pyrolysis temperatures from 400°C to 700 °C. Williams et al. [55] found that when the pyrolysis temperature increased from 450 °C to 600 °C, the aromatic content increased and the aliphatic content decreased. At 475 °C, the maximum oil yield was 58.2 wt%, while at 600 °C, the oil yield decreased to 53.1 wt%.

Miranda et al. [60] studied the dynamic residues in the pyrolysis processes of NR, BR, and SBR components and found that the lower temperature (<390 °C) was conducive to the formation of olefins, while the higher temperature was conducive to the formation of aromatics. Rodriguez et al. [42] pyrolyzed automobile tires in autoclaves filled with nitrogen at 300–700 °C and found that when the temperature was above 500 °C, the quantity and quality of pyrolysis products did not change. When the temperature was lower than 500 °C, the main compounds in pyrolysis products were isoprene and limonene. Menares et al. [18] found that when the temperature was above 600 °C, it was conducive to the formation of single aromatics and gases. Laresgoiti et al. [61] conducted pyrolysis of the entire automobile tire in a high-pressure kettle filled with inert gas at 400–700 °C and found that carbon oxides ( CO_x_) and lighter gases at high temperature were generated due to secondary pyrolysis of inorganic components and products. Yazdani et al. [62] pyrolyzed WTs in a rotary kiln filled with nitrogen (N_2_), and the pyrolysis temperature was 400–1050 °C. The results showed that the highest yield of pyrolysis oil was 44 wt% at 550 °C.

Although the pyrolysis temperature of WTs is the main experimental control variable, the experimental results are also directly affected by other important factors, such as pressure, pyrolysis equipment, heating rate, catalyst, residence time, etc. Pyrolysis oil production may decrease or increase at certain temperatures and is also associated with the addition of specific catalysts, which may also contribute to increased gas production or oil production. Next, the effects of different types of catalysts on the pyrolysis process and the products are described in detail.

### 3.2. Catalysts

The pyrolysis reaction takes place at a certain temperature, which is called the critical temperature of pyrolysis. The critical temperature of the maximum molecular chain of rubber is about 380 °C, and when the pyrolysis temperature is higher than 420 °C, it is called high-temperature pyrolysis. Polycyclic aromatic hydrocarbons (PAH) (commonly known as dioxins), which are readily produced by high-temperature pyrolysis, are powerful carcinogens. Generally speaking, the higher the pyrolysis temperature, the more noncondensable combustible gas produced by pyrolysis, the lower the oil yield and—at the same time—the higher the proportion of aromatic hydrocarbons in oil products, resulting in the quality of oil products and carbon black decreased. The output and quality of pyrolysis oil and gas obtained from pyrolysis of WTs not only depend on the type of tires but also on the type and conditions of pyrolysis process [63]. Catalysts have great influence on tire pyrolysis. In general, compared with noncatalytic pyrolysis, catalytic pyrolysis is beneficial in increasing gas production and reducing pyrolysis oil, but the use of catalysts has little effect on coke yield.

Catalysts can accelerate the reaction rate and reduce the reaction activation energy and the wastage of the energy used in pyrolysis. Additionally, their own chemical properties do not change before and after reaction, and they themselves have no consumption, so they can be used repeatedly. At the same time, they can also reduce the requirements for pyrolysis equipment. In addition, for complex reaction, catalytic pyrolysis can choose to speed up the main reaction rate, restrain side effects, and improve the yield of the target products. In the catalytic pyrolysis of WTs, the material, aperture, structure, performance, and stability of the catalysts have great influence on the pyrolysis process. Catalysts with large pore size and low Si-Al ratio have better extraction effect on aromatics, but the use of such catalysts can reduce the output of pyrolysis oil [7].

The low-temperature catalytic pyrolysis of WT is a pyrolysis reaction at a temperature slightly higher than the critical pyrolysis temperature. The presence of a catalyst reduces the reaction temperature and time required for pyrolyzing. Most important is that the use of catalysts can improve the quality of pyrolysis oil and carbon black basically without PAHs in pyrolysis products and with no harm to the environment. Zhang et al. [64] reported that the use of alkaline additives and catalysts reduced the reaction temperature. Miranda [60] believed that the activation energy of rubber pyrolysis was 127.4–176.0 kJ/mol and that the use of different types of catalysts would reduce the activation energy of WT pyrolysis to different degrees. Another advantage of catalytic pyrolysis over thermal pyrolysis was that the catalytic pyrolysis of the polymerization chain resulted in a narrower variety of products [65]. The results of GC-MS showed that there were up to 93.3% mixed aromatic compounds and a small amount of aliphatic hydrocarbons in the thermal pyrolysis oil. The use of catalysts reduced the concentration of aromatic compounds in pyrolysis oil [66]. The use of fluid catalytic cracking (FCC) catalysts made it less likely to produce aromatic compounds [38]. Miandad et al. [66] found that in the absence of a catalyst, the yield of liquid oil could reach 40%; under the catalyzed conditions of activated alumina, activated calcium hydroxide, natural zeolite, and synthetic zeolite, the yields of pyrolysis oil were 32 wt.%, 26 wt.%, 22 wt.%, and 20 wt.%, respectively. Table 2 shows the content of pyrolysis products when different types of catalysts are used for waste tire pyrolysis.

#### 3.2.1. Zeolite Catalysts

Among various catalysts used in WT pyrolysis, zeolites are the most common. Zeolite catalysts are popular because of their acidity and unique pore structure (there are six main types of zeolites). Zeolite can be used as a catalyst alone or can be mixed with precious metals to form a synthetic catalyst containing a variety of material components. Different catalysts have different functions, and some may even allow several chemical mechanisms to occur simultaneously. Zeolite catalysts can be used in the pyrolysis of WTs to prepare compounds with high aromatic content, which has great application value. Next, the influence and mechanism of some zeolite catalysts on the composition and content of pyrolysis products are summarized.

In general, the use of acid catalysts can reduce the yield of liquid oil and increase the yield of gas. Ultra-stable Y zeolite (USY) is often used as catalyst for upgrading chemical products due to its high activity and stability. Williams et al. [71,72] found that the gas yield obtained from USY and HZSM-5 catalyzed pyrolysis increased. Wang et al. [73] studied the effects of USY zeolite catalysts with different SiO_2_/Al_2_O_3_ molar ratios on the formation of aromatic hydrocarbons. The results showed that USY with SiO_2_/Al_2_O_3_ ratio of 5.3 was more conducive to the formation of aromatic hydrocarbons, and USY with a high SiO_2_/Al_2_O_3_ molar ratio (11.5) was more conducive to the formation of olefins. Vichaphund et al. [74] adopted the HZSM-5 zeolite processed through three different modes as catalysts and carried out the catalytic pyrolysis of waste rubber at the pyrolysis temperature of 500 °C. It was found that the increase in HZSM-5 catalyst content significantly promoted the formation of aromatic hydrocarbon compounds—especially an increase in the yield of benzene, toluene, and xylene—but the content of nonaromatic components in the pyrolysis products significantly decreased. Santos et al. [75] found that in the catalytic pyrolysis of waste rubber, USY zeolite had higher surface area and larger pore size than HZSM-5 zeolite, so the usage of USY zeolite was more conducive to the generation of aromatic hydrocarbon in catalytic pyrolysis. Manchantrarat et al. [76] used various zeolites for catalytic pyrolysis of waste rubber. They found that Y-type zeolites significantly increased the yields of saturated and monoaromatic hydrocarbons and reduced the yields of diaromatic, polyaromatic, and polar aromatic hydrocarbons. Boxiong et al. [67,77] found that through the usage of the molecular sieve USY catalyst and the HZSM-5 catalyst, high concentrations of monocycle aromatics—such as benzene, toluene, and xylene—could be prepared, and at the same time, the oil yield could be reduced and the gas phase yield improved. Higher concentrations of aromatic hydrocarbons could be obtained from the catalytic pyrolysis with the USY of waste tires, so USY was considered to be a more suitable catalyst for the preparation of raw chemical materials. Other researchers [70] found that the concentrations of benzene, toluene, and xylene in the pyrolysis products was high, while the concentration of limonene was decreased when USY zeolite catalyst was used. The reason was that the larger the USY pore size, the lower the acidity and catalytic activity and the higher the selectivity for limonene to decompose into aromatic hydrocarbons. Li et al. [69] found that the catalytic pyrolysis gas yield with different catalysts was also different, and the order of gas yield was roughly SAPO-11 > USY > Hβ > HZSM-5 > HZSM-22 > non-catalyst. The use of HZSM-5 catalyst resulted in the highest yield of pyrolysis oil (55.65%), while the use of SAPO-11 catalyst resulted in the highest gas production (10.45%) and the lowest carbonization rate (34.43%).

Metal or metal oxide doping can enhance the activity of a catalyst, so this method is increasingly used in the catalytic pyrolysis of WTs. Hijazi et al. [78] studied the catalytic pyrolysis of WTs catalyzed by Hβ and Pd/Hβ zeolite. The results showed that the gas yield of noncatalytic pyrolysis was 20% and that the gas yield increased to 28% and 37% by adding Hβ and Pd/Hβ, respectively. The stronger catalytic activity of Pd/Hβ was due to the dehydro-hydrogenation reaction caused by Pd metal sites. The results of GC-MS showed that under the action of Pd/Hβ catalyst, the composition of oil had significantly shifted towards a low carbon number (C_9_–C_13_), and the carbon number of hydrocarbons in pyrolysis oil decreased from diesel (C_12_–C_20_) to the narrow range of gasoline and naphtha (C_9_–C_12_). Additionally, Hijazi et al. [79] also prepared polyphase photocatalysts by TiO_2_ doped with metal Pd, Pt and metal oxide Bi_2_O_3_/SiO_2_, respectively. The results of WT pyrolysis with and without catalysts at 550–570 °C showed that the gas production rate of noncatalytic pyrolysis was 20%, and that of pyrolysis catalyzed by TiO_2_ was 27%. It was also found that doping TiO_2_ with precious metals and metal oxide would improve the catalytic capacity of catalysts. The gas production of pyrolysis catalyzed by Pd-Pt/TiO_2_ and Pd/TiO_2_ has maximum values of 40% and 41%, respectively. Metal doping changed the morphology of TiO_2_, resulting in the increase in nano grain size, pore volume and specific surface area. Pd can induce hydrogenation/dehydrogenation reaction. By converting alkanes to olefins, the olefins are isomerized and cracked at acidic sites near zeolite so as to help improve the catalytic activity. Basagiannis et al. [80] found that ruthenium (Ru) can also be doped with zeolite, which helped to increase catalytic activity, reduce pyrolysis temperature, and increase hydrogen production rate. Zeolite catalysts with noble metal carriers could catalyze the hydrogenation of raw materials, enhance the pyrolysis efficiency of WTs, and promote the removal of heteroatoms (sulfur and oxygen) [81]. Yu et al. [27] modified zeolite by doping copper and found that the use of strong acid sites could help reduce the sulfur content of pyrolysis products and improve oil quality. The Y-type zeolite catalyst has a high coking rate. Doping cerium into the zeolite structure through ion exchange technology to replace protons can change the activity and pore properties of the catalyst and finally reduce the formation of coke in pyrolysis reaction. The coking rate of large porous Y-type zeolite decreased from 8.1% to 5.7% after cerium ion exchange.

Mesoporous MCM-41 zeolite inhibited the formation of polycyclic aromatic and polar aromatic, and promoted the formation of monoaromatic and saturated hydrocarbons [82]. Khalil et al. [83] used a two-stage fixed bed for the catalytic pyrolysis of waste rubber and found that microporous zeolite catalysts increased the yield of aromatic compounds by 23.7% and that mesoporous McM-41 catalysts increased it by 18.7%. MCM-48 is a mesoporous material with a cubic crystal structure and has a better catalytic effect. The content of light olefin produced by Ru/McM-48 catalyst was twice that of noncatalytic cracking, while the proportion of light oil also increased, which improved the quality of oil products. In addition, the sulfur content of polyaromatic compounds and polar aromatic compounds in pyrolysis oil decreased [53]. Dũng et al. [82] studied the MCM-41 and Ru/MCM-41 in the catalytic activity of waste tire pyrolysis. The results showed that gas production increased while liquid production decreased, and lighter oil was produced after adding the two catalysts. The yield of light olefin catalyzed by Ru/MCM-41 was four times that of noncatalytic pyrolysis.

#### 3.2.2. Other Catalysts

Apart from zeolites, some natural catalysts or some acid/alkali additives were also used in the catalytic pyrolysis of WTs. Miandad et al. [66] pyrolyzed WTs in a 20 L small semi-industrial scale reactor. The results of GC-MS confirmed that the aromatic compound content in the oil prepared from non-catalyst pyrolysis was as high as 93.3%. After the pyrolysis process was catalyzed by active calcium hydroxide, natural zeolite, and activated alumina, the concentrations of aromatic compounds in the obtained pyrolysis oil were reduced to 60.9%, 71.0%, and 84.6%, respectively. Ibrahim et al. [84] added 5% nickel to the calcined dolomite as a catalyst for the catalytic cracking of WTs; the gas yield increased from 30.3 to 49.1 wt.%, and the hydrogen yield doubled. At this time, the coke deposited on the surface of the catalyst reached a minimum of 0.9 wt.%. Itkarnka et al. [85] used HNO_3_-treated pyrolysis carbides of waste rubber as catalysts during waste rubber pyrolysis. Acid-treated carbides had higher acidity and larger surface area and pore size, and the use of this catalyst for catalytic pyrolysis of waste rubber increased gas production and promoted greater pyrolysis activity. In addition, Williams et al. [86] synthesized carbon nanotubes and hydrogen from the pyrolysis products using Ni/Al_2_O_3_ as a catalyst in a two-stage fixed bed reactor, and the results showed that at the tire:catalyst weight ratio of 1:1, the highest yield of filamentous carbons was produced at 253.7 mg/g tire.

## 4. Properties and Applications of Pyrolysis Products

**Figure 7:**
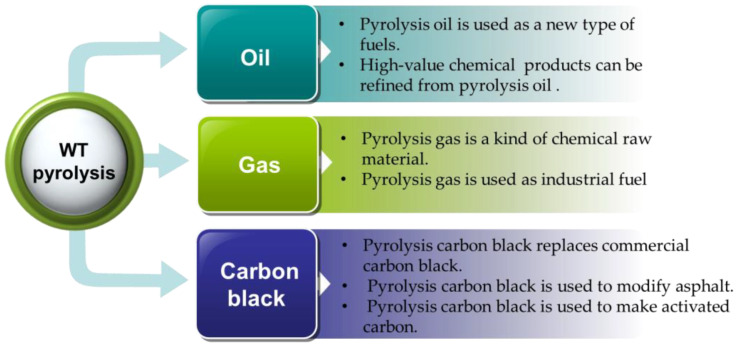
Applications of WT pyrolysis products.

Through WT pyrolysis, three kinds of valuable products can be obtained, and their applications are rather wide, as shown in Figure 7.

### 4.1. High Value Pyrolysis Oil

Pyrolysis oil is the liquid product condensed from the volatile fraction of WT pyrolysis, which is a black opaque liquid with pungent smell. It is a very complex mixture. According to the experimental verification, there are more than 100 compounds in it, which can be converted into gasoline (C_5_–C_10_), diesel (C_14_–C_18_), and heavy oil (>C_18_) after purification. The pyrolysis oil contains aliphatic, aromatic, heteroatom, and polar components. Among them, aromatic hydrocarbons—including benzene, toluene, xylene, styrene, limonene, ninhydrin, and their alkylated homologues and 2–5 ring polycyclic aromatic hydrocarbons—account for a large proportion (about 62.4%). Aliphatic hydrocarbons account for a relatively small proportion(about 31.6%). The main aliphatic compounds are alkanes, the straight-chain hydrocarbons formed from C_6_–C_37_, and the olefin at a lower concentration [87]. The main components of aromatic hydrocarbons are monocyclic aromatic hydrocarbons, which account for about 43–58% of the total weight of aromatic hydrocarbons. The main components of aliphatic hydrocarbon include light aliphatic hydrocarbon and heavy aliphatic hydrocarbon. The composition of light aliphatic hydrocarbons is mainly olefins, while the composition of heavy aliphatic hydrocarbons is mainly n-alkanes. In the pyrolysis process, the chemical bonds of rubber are thermally decomposed under inert gases, and the pyrolysis products range from light alkane gases to heavy complex aromatic hydrocarbons. The composition of WT pyrolysis oil is not invariable, which is mainly affected by the raw material composition of WTs, pyrolysis temperature, pressure in the pyrolysis reactors, residence time, and so on [88,89,90,91].

Dai et al. [92] found that the pyrolysis oil obtained from WTs in a circulating fluidized bed contained alkane (26.77 wt%), aromatics (42.09 wt%), non-hydrocarbons (26.64 wt%), and asphalt (4.05 wt%). Nisar et al. [28] analyzed the pyrolysis products of WTs via GC-MS, and the results showed that the hydrocarbon carbon chain of noncondensable gas and liquid compounds was mainly distributed in C_1_–C_5_ and C_16_–C_19_, respectively. Laresgoiti et al. [93] found that pyrolysis oil contained a complex mixture of C_6_-C_24_ organic compounds, consisting mainly of aromatic compounds (53.4–74.8%), some nitrides (2.47–3.5%), and some oxygen compounds (2.29–4.85%), with a sulfur content of about 1.0–1.4%.

#### 4.1.1. Pyrolysis Oil Is Used as a New Type of Fuel

Pyrolysis oil is highly acidic, with high sulfur content and low thermal stability. In addition, other physical properties, such as viscosity and the flash point of pyrolysis oil, make it unable to burn directly or replace engine fuel. The sulfur content of pyrolysis oil is generally 1.0–2 wt%, while that of general commercial diesel oil is less than 0.05 wt%. The components of pyrolysis oil are complex, and the existence of low-boiling-point compounds leads to the flash point of pyrolysis oil generally being lower than 30 °C. The lower the flash point, the lower the safety. The pyrolysis oil must be further refined to improve its performance. As shown in Table 3, the physical properties of the pyrolysis oil produced under different conditions are also different.

WT pyrolysis oil must be treated before it can be used as fuel oil. Currently, the commonly used treatment methods include hydrotreating, catalytic treatment, fractionation, copyrolysis, activated carbon adsorption, etc. [97,98]. Costa et al. [99] extracted light fuel fraction from tire pyrolysis oil via steam distillation. Light fuel fraction (LFF) is a light-yellow translucent liquid with a specific gravity of 0.76 g/cm^3^ and a dynamic viscosity of 0.4 MPa.s at 20 °C. The light component is mainly composed of benzene compounds (62.06%), ethyl benzene (14.84%), and methyl benzene derivatives (13.02%). It was found that the components of light fuel are very similar to those of gasoline extracted from petroleum, and it was feasible to replace traditional gasoline with light fuel. According to Miandad et al. [66], the physical properties of waste rubber pyrolysis oil, such as high heat value (HHV) (42–43.5 MJ/kg), kinematic viscosity (1.9 cSt), density (0.9 g/cm^3^), pour point (−2 °C), and flash point (27 °C), were close to the standard value of conventional diesel oil. Moreover, the liquid oil had higher calorific value, which is the same as conventional diesel oil (42.7 MJ/kg). WT pyrolysis oil contains large amounts of sulfide (1.15 wt%), so it is not suitable for internal combustion engines. Jantaraksa et al. [100] found that the WT pyrolysis oil could be improved by catalytic hydrodesulfurization of cobalt molybdenum supported by Molybdenum (Mo), nickel molybdenum (NiMo) or alumina oxide (AL_2_O_3_). The reaction took 30 min, and the maximum desulfurization rate is 87.8%. The calorific value of hydrolyzed pyrolysis oil (44 MJ/kg) is similar to that of commercial diesel fuel (45 MJ/kg) and gasoline fuel (47 MJ/kg). The results of fuel properties show that the properties of pyrolysis oil are basically the same as those of industrial diesel oil. However, to obtain high-quality fuels, further treatment such as hydrogenation, desulfurization or the use of catalysts are required. The treated pyrolysis oil can be mixed with diesel or other fuels in different proportions to meet the standard and serve as a fossil fuel substitute for motor vehicles. The treated pyrolysis oil can also provide high-calorific fuel for large fuel equipment, such as internal combustion engine boilers and heating furnaces. In summary, pyrolysis oil has a strong potential to replace traditional fuels.

#### 4.1.2. Refining High-Value Chemical Products from Pyrolysis Oil

After distillation, pyrolysis oil can be divided into three parts: naphtha fraction, medium fraction, and heavy fraction. Distillation can make pyrolysis oil more valuable for use. Li et al. [92] found that pyrolysis oil produced from waste tire pyrolysis in a rotary kiln contained 39.2–42.3 wt% of light naphtha (below 200 °C), a medium fractionation of 32.4–33.2 wt% (200–350 °C), and a heavy fractionation of 25.5–28.5 wt% (above 350 °C). Researchers generally found that higher temperatures promoted the formation of lighter pyrolysis oil, such as gasoline and kerosene. Table 4 reflects the fractions of pyrolysis oil at different pyrolysis temperatures.

Naphtha fractions obtained from the distillation of pyrolysis oil can be used to extract valuable chemical products, such as benzene, toluene, xylene, limonene, and phenolic compounds [103]. Among them, limonene has high economic value and high yield and can be used as solvent and aromatic agent. In the process of waste rubber pyrolysis, the C-C bonds in the double bonds of polyisoprene tend to break to form allyl radicals. limonene is generated after the cyclization of allyl radicals. Limonene is unstable and decomposes easily at high temperatures. Limonene production is increased at lower cracking pressures and shorter residence times. Limonene yield decreases with the increase in temperature, with the highest yield at 400–500 °C [104]. Other pyrolysis oil distillates are also important and widely used. Benzene is used in drugs, surfactants, and dyes. Toluene is used in the production of pesticides, dyes, surfactants, and solvents. O-xylene is used in the production of plasticizers, dyes, and pigments. M-xylene derivatives are used in the fiber industry, and p-xylene derivatives are used in the production of polyester fibers [93]. It can be seen from the above that the chemical products extracted from pyrolysis oil are of high economic value and are widely used in industry.

### 4.2. Properties and Applications of Pyrolysis Gas

Pyrolysis gas is the gaseous product after rubber pyrolysis, and it is a noncondensable gas formed in the pyrolysis process. The gaseous products of WT pyrolysis are alkanes (C_1_–C_4_), olefins (C_1_–C_4_), hydrogen (H_2_), carbon monoxide (CO), carbon dioxide (CO_2_), and sulfur and nitrogen compounds in low concentrations [105,106,107,108,109,110]. The contents of H_2_ (30.4%) and methane (CH_4_) (23.3%) is highest in the pyrolysis gas, with a calorimetric value of about 38.5 MJ/Nm^3^ [111].

Based on the analysis and determination of GC, the main components of pyrolysis gas are CH_4_, ethane (C_2_H_6_), ethylene (C_2_H_4_), propylene (C_3_H_6_), propane (C_3_H_8_), acetylene (C_2_H_2_), butane (C_4_H_10_), butane (C_4_H_8_), 1,3-butadiene (C_4_H_6_), pentane (C_5_H_12_), benzene (C_6_H_6_), toluene (C_7_H_8_), xylene (C_8_H_10_), H_2_, nitrogen (N_2_), CO, CO_2_, and hydrogen sulfide (H_2_S) [64].

The composition of pyrolysis gas mainly depends on the composition of raw materials for tire preparation, such as SBR, NR, nitrile rubber, neoprene rubber, polybutadiene rubber, and so on. The gas composition is also related to pyrolysis temperature, pressure, etc. The long rubber polymer chain is broken at high temperatures to produce short-chain gas products. With the increase in temperature, the pyrolysis products will have secondary reactions, producing lighter gas and higher contents of hydrogen, methane and C_1_–C_4_ hydrocarbons. Wei et al. [32] studied the production process of pyrolysis gas by combining RMD and TG-FTIR. The results showed that CH_3_ separated from the main chain extracted H from other molecules to produce CH_4_, while C_2_H_4_ was mainly produced by the C=C bond fracture on the long chain. Other small gas molecules are produced by the decomposition of large alkenes or low-activity free radicals. Xu et al. [33] found that the generation of CH_4_ and C_2_H_4_ mainly occurred in the range of 350–600 °C. The formation of CH_4_ is generally attributed to the decomposition of -CH_3_ and -CH_2_ on aliphatic hydrocarbons. The formation of C_2_H_4_ is mainly attributed to the decomposition reaction of free radicals resulting from the bond-breaking of the olefin ring and aromatic ring on the straight chain and the side chain [112]. The total gas production generated by pyrolysis of tires will increase with the increase in pyrolysis temperature. The higher the temperature, the higher the gas production and the lower the oil production [52,113]. Kaminsky et al. [52] found that as pyrolysis temperature increased from 598 °C to 700 °C, pyrolysis yield increased from 20 wt% to 33 wt%, the hydrogen yield increased from 0.59 wt% to 1.1 wt%, the CH_4_ content increased from 2.9 wt% to 6.9 wt%, C_2_ hydrocarbons increased from 2.8 wt% to 5.8 wt%, and C_3_ hydrocarbons increased from 2.96 wt% to 5.03 wt%. 

Pyrolysis gas contains valuable olefins, such as ethylene and propylene, which can be purified as an important chemical raw material. The content of pyrolysis gas and the valuable olefins can be increased through controlling factors, such as pyrolysis temperature [114] and catalysts [115], during the pyrolysis process. Li et al. [116] found that high H_2_ yield could be obtained by pyrolysis of WTs with Ni supported on activated carbon. Luo et al. [117] added blast furnace slag into the reactor during the process of WT pyrolysis and found that blast furnace slag could act as a dehydrogenation catalyst, significantly increasing the production of gas products and the contents of H_2_ and CO in the gas. Hydrogen can be purified through the hydrogen production process when hydrogen content is high [118,119,120]. Kuznetsov et al. [121] conducted plasma gasification of tires at a maximum temperature of 2073 K and used calcium oxide as catalyst to improve the production of hydrogen, and the hydrogen content increased from 58% to 99%. Portofino et al. [120] conducted catalytic pyrolysis of WTs in a rotary kiln and found that when the pyrolysis temperature was 823 K, the concentration of CH_4_ (42%) was the highest—followed by that of H_2_ (30%)—and when the pyrolysis temperature was 1023 K, the concentration of H_2_ (57%) was the highest—followed by that of CH_4_ (21%).

The pyrolysis gas has stable physical properties and low sulfur content, and the calorific value is equivalent to that of natural gas, which can reach 6390–10,230 kJ/kg. Raman et al. [119] obtained a maximum pyrolysis gas yield of 0.76 Nm^3^/kg at 1100 K, and the gas calorific value was 39.6 MJ/Nm^3^. Galvagno et al. [51] obtained the pyrolysis gas using an FBR at 550 and 680 °C, and the calorific values were 22 and 29 MJ/kg, respectively. Laresgoiti et al. [61] found that the calorific value of pyrolysis gas prepared from the autoclave reactor under N_2_ atmosphere was higher. When the pyrolysis temperature was 400 °C, the gas calorific value was 81 MJ/m^3^, and when the pyrolysis temperature was 700 °C, the gas calorific value was 69.5 MJ/m^3^. 

At present, pyrolysis gas is most commonly used in supplying the heat required by the pyrolysis process, and many self-sufficient pyrolysis devices have been developed successfully [20,51,61,122]. Furthermore, after simple treatment, pyrolysis gas can be used as industrial fuel for heating various large fuel equipment or factories, which has good market development prospects.

### 4.3. Properties and Applications of Pyrolysis Carbon Black

The solid products of WT pyrolysis are carbon black and ash composed of inorganic compounds, which account for about 35–40 wt% of the total weight of WTs. The carbon black accounts for 80–90 wt% of the solid products [6]. The main elements of pyrolysis carbon black are carbon (81.5–82.8 wt%), hydrogen (0.32–1.0 wt%), sulfur (1.7–3.3 wt%), and nitrogen (0.2–0.5 wt%) [110,123]. Meanwhile, there are still some other pollutants in the solid products, such as dust, heavy metals, volatile substances, and trace oil [124].

Carbon black is an important reinforcement filler of tires and plays the functions of coloring, reinforcing, conducting electricity, thermal conducting, anti-ultraviolet rays, and other functions in rubber refining and has an important influence on the physical and mechanical properties and processing technology of rubber compounds. Pyrolysis carbon black is a recyclable resource with high economic value, but it cannot achieve the reinforcing effect of industrial carbon black. The low-boiling-point material content and ash content of tire pyrolysis carbon black are higher than those of commercial carbon black (N550). High ash content and the presence of impurities have adverse effects on the curing characteristics, cross-linking density, and mechanical properties of carbon black [125]. There are functional groups on the carbon black’s surface, such as carboxyl, phenols, and ketones, which enhance the carbon black surface activity and improve the strength of the interaction of carbon black and rubber. However, the ash attached to the carbon black surface covers active sites of the carbon black surface [126], hindering the interaction between the carbon black and the polymer, affecting the reinforcement, which becomes the main obstacle to the recovery of pyrolysis black carbon. Tang [127] prepared carbon black by plasma pyrolysis of tire particles and compared it with commercial carbon black. It was found that the surface area of pyrolytic carbon black was 64.8 m^2^/g and that the ash content was 15.14 wt%, while the surface area of commercial carbon black (N330) was 80 m^2^/g, and the ash content was 0.4 wt%. Compared with other pyrolysis equipment, the surface area of pyrolysis carbon black produced by FBR was lower [96]. Meanwhile, pyrolysis pressure and temperature also have certain effects on the quality of pyrolysis carbon black. Sahouli et al. [128] compared the surface chemistry and morphology characteristics of carbon black recovered under different pyrolysis conditions and found that the surface characteristics of carbon black recovered via low-pressure pyrolysis were similar to those of the corresponding raw material carbon black. It was found that when the pyrolysis temperature was 425 °C, the specific surface area of pyrolysis carbon black was 46.5 m^2^/g, and when the pyrolysis temperature rose to 600 °C, the specific surface area of pyrolysis carbon black significantly increased to 116.30 m^2^/g. The reason was that with the increase in pyrolysis temperature, the hydrocarbons on the pyrolysis carbon surface were decomposed, reducing the coking on the carbon black surface [36]. In addition to the specific surface area, pyrolysis temperature also has a certain effect on some other important parameters of pyrolysis carbon black, as shown in Table 5.

According to the data in Table 5, the unprocessed pyrolysis carbon black cannot be used in rubber processing. At present, the pyrolysis carbon black of WT pyrolysis is mainly applied in the following aspects: Firstly, when the particle size and surface active structures of pyrolysis carbon black meet the requirements after treatment, it can be used as a substitute for commercial carbon black and as a reinforcing agent and filler for rubber products. Secondly, it can be added to asphalt for asphalt modification. In the third aspect, activated carbon is prepared via physical and chemical methods. Pyrolysis carbon black is not used in large quantities in other applications, such as in coatings for the automotive industry—where it is protected from ultraviolet light—or in inks—where it is used to provide pigmentation.

#### 4.3.1. Pyrolysis Carbon Black Replaces Commercial Carbon Black

The solid carbon produced by waste tire pyrolysis contains some high temperature pyrolysis products of additives. Elemental analysis shows that the solid residue contains 71 wt% C and some other elements—such as Fe, S, Zn, etc.—as well as a large amount of ash [132]. Therefore, solid carbon is usually treated with acid and alkali to remove inorganic elements and reduce ash and sulfur content. Jitkarnka et al. [85] found that acid treatment increased the surface area and pore size of carbon. The increase in total acidity of carbonates is due to the enhancement of the surface carboxyl (-COOH) groups. In addition, HNO_3_ treatment can make coke demineralize and significantly reduce the content of sulfur compounds in coke. Differently from other acids, HNO_3_ can maintain the pore structure of coke so that the coke has a great advantage in dust removal. HNO_3_ and NaOH were used to chemically leach the solid carbon black generated in the continuous pyrolysis process. The ratio of reagent/pyrolysis carbon was 10 mL/g, and 4.9 wt% of the ash content could be removed by soaking at 60 °C for 60 min [130]. Chaal et al. [129] acid pickled and alkaline washed cracked carbon black with H_2_SO_4_ and NaOH, respectively, under vacuum conditions. The results showed that: after acid–base treatment, the content of C element in pyrolysis carbon black increased significantly, and the ash content of pyrolysis carbon black decreased to 3.1 wt% (14.6 wt% before treatment). The pyrolysis carbon black was pickled and alkaline washed to remove ash on its surface and then reacted with stearic acid. The carboxyl group of stearic acid could esterify the hydroxyl group on the surface of carbon black, producing a long-chain alkyl group at one end of carbon black molecule, which enhanced its bonding with rubber. Tian et al. [133] prepared pyrolysis carbon black/rubber composites based on a novel approach called atomization dispersion and high temperature sputtering drying (ADSD method) (as shown in Figure 4). Before the ADSD mixing method, the pretreatment of pyrolysis carbon black had been performed, i.e., the pyrolysis carbon black was mechanically ground. Through the ADSD method, the uniform dispersion and instantaneous drying of the pyrolysis carbon black in the latex were realized. The performance of this carbon black/rubber composite was even better than that of composites prepared via traditional methods. In a word, after the improvement of surface structure of pyrolysis carbon black, it can be used in rubber products instead of industrial carbon black, which is of great significance for the recovery and utilization of waste tire pyrolysis solid products.

#### 4.3.2. Pyrolysis Carbon Is Used to Modify Asphalt

Studies have shown that the addition of commercial-grade carbon black to asphalt can effectively improve the photo-oxidation aging rate of asphalt [134,135]. The addition of carbon black obtained from waste rubber pyrolysis to asphalt also had a positive effect on the rheological properties of asphalt [136,137]. Feng et al. [138] added the treated waste tire pyrolysis carbon black to the asphalt for the asphalt modification treatment, and the results showed that if the pyrolysis carbon black content was not more than 10%, the pyrolysis carbon black can significantly improve its high temperature performance, the ability of permanent deformation at high temperature, can obviously improve the thermal aging and photo-oxidation aging. Modified asphalt can be used in road construction, which is of great significance to improve the pavement performance.

#### 4.3.3. Pyrolysis Carbon Is Used to Make Activated Carbon

Pyrolysis carbon can also be used as an adsorbent, but due to its low surface area, which is approximately 30–90 m^2^/g, it is required to be activated to increase its porosity and surface area [139]. The porous activated carbon made from WT pyrolysis carbon black has a high adsorption capacity and can also remove various pollutants, such as heavy metals, dyes, pesticides, and other pollutants from water media [140]. Shah et al. [141] found that the adsorption capacity of acid treated pyrolysis carbon black was even higher than that of commercial activated carbon.

Traditional activated carbon preparation is divided into two methods: physical and chemical activation. During physical activation, the carbon material reacts with steam, carbon dioxide, or other oxidizing gases at a high temperature to make the disordered carbon in the carbon material partially oxidized and etched into pores, forming a developed microporous structure inside the material. It had been experimentally confirmed that the surface area of activated carbon produced via steam activation method is 20% higher than that produced by the carbon dioxide activation method [87]. Chemical activation is the preparation of activated carbon by mixing an appropriate proportion of activator (H_2_SO_4_, KOH, H_3_PO_4_) with raw materials and directly activating it [142]. Chemical activation has many advantages over physical activation, such as low activation temperature, high yield, high surface area, and large pore volume. Rambau [139] adopted a mechanochemical method to activate the pyrolysis carbon. Before activation, the pyrolysis carbon was compacted by mechanical methods with activator to increase its active site, and then the pyrolysis carbon was treated with water, HF, and HNO_3_. According to data analysis, the surface area of pyrolysis carbon was the largest after washing by HNO_3_, which was 955.20 m^2^/g. The purpose of acid treatment was to remove inorganic elements and generate more pores on the surface of carbon, thus increasing its surface area. Acosta et al. [143] activated pyrolysis carbon black with KOH in a temperature range of 600–800 °C to prepare activated carbon. The surface area of activated carbon prepared was up to 814 m^2^/g, and the surface area, micro porosity, and medium porosity were greatly improved. Gupta et al. [144] used microwave-assisted processing prepared high quality activated carbon from pyrolysis carbon black. Rahmani et al. [145] investigated that the activated carbon prepared from pyrolysis carbon black had a good adsorption effect on lead metal cations from lead-containing aqueous solutions. Trubetskaya et al. [146] used pyrolysis carbon black for the cleaning of wastewater and removed up to 95% of phenol and chloride. Therefore, after necessary means, pyrolysis carbon black is expected to replace commercial activated carbon [36,147,148].

## 5. Conclusions

The WT pyrolysis technology has been widely studied. At present, catalytic pyrolysis is the best treatment method for WTs. Through this method, a large number of WTs can be thoroughly treated to reduce the environmental pollution and obtain high-value pyrolysis products. WT pyrolysis includes physical and chemical reaction, that is, condensation, polymerization, depolymerization, and aromatization of rubber occur at an appropriate temperature. Usually, the progress of WT pyrolysis is divided into the following four steps: first, water vapor and additives decompose; NR begins to decompose at about 300 °C; around 400 °C, SR begins to decompose; at 500 °C, pyrolysis is basically completed. The yield and composition of pyrolysis products depend largely on temperature and catalysts. With the increase in pyrolysis temperature, the content of pyrolysis gas increases and the content of pyrolysis oil decreases. The higher the pyrolysis temperature, the faster the secondary reaction will occur, leading to an increase in the content of aromatic compounds. Zeolite catalysts are beneficial to the generation of aromatic hydrocarbons (especially monocyclic aromatic hydrocarbons, such as benzene, toluene, and xylene). At the same time, the use of zeolite catalysts makes the yield of oil reduce and the gas production increased. Pyrolysis oil and gas obtained from WT pyrolysis have a high calorific value (35–44 MJ/kg). After being treated, pyrolysis oil can be used as alternative fuel for engines or factory fuel. After distillation, the pyrolysis oil also can be purified as chemical raw materials, such as phenylxylene, toluene, and limonene. The pyrolysis carbon can be used as commercial carbon black or activated carbon or used in asphalt modification. Pyrolysis gas can be used directly for industrial fuel and hydrogen production. In a word, WTs can be converted into valuable products via pyrolysis. At present, the pyrolysis process and continuous equipment are not perfect, and the profitability of the pyrolysis of WTs depends on the main products obtained. Therefore, the pyrolysis products should be further refined and processed to produce products of higher value so as to make the pyrolysis process more economically feasible.

The future research on WT pyrolysis should focus on the influence mechanism of reaction conditions on reaction path so that the pyrolysis products can achieve directional regulation. More novel methods of tests and experiments should be applied to the research of WT pyrolysis so that the pyrolysis mechanism can be explored in detail. In addition, researchers should also focus on the relationship between product selectivity and pollutant reduction. Only considering various factors, the environmental and economic benefits of WT pyrolysis can be further improved, and its degree of industrialization can be continuously expanded.

## Figures and Tables

**Table 1:** Oil production at different pyrolysis temperatures.

Ref.	Temperature (°C)	Oil (wt%)	Pressure (Pa)
[55]	450	58.1	101,325
	525	56.9	
	600	53.1	
[56]	450	49	101,325
	500	45	
	600	40	
[41]	400	36	101,325
	500	44	
	600	45	
[57]	350	30	101,325
	450	33	
	550	38	
[58]	450	43	8000–9000
	550	44.6	
	650	42.9	
[43]	500	55.4	
	600	52.2	
	700	36.6	

**Table 2:** Distribution of pyrolysis products under different catalysts.

Temperature (°C)	Catalysts	Yield (wt%)			Ref.
		Oil	Char	Gas	
450	-	50.47	36.47	13.06	[67]
500	-	51.98	36.09	11.92	
550	-	52.61	35.69	11.70	
600	-	54.10	36.30	9.61	
500	Ca(OH)_2_	40	48	12	[68]
500	Na_2_CO_3_	47.8	37.6	14.6	[64]
500	ZSM-5	55.6	37.6	6.5	[69]
500	USY	53.5	36.5	10	
450	HZSM-5	50.2	33.1	16.7	[70]
450	HY	54.9	33	12.1	
450	Hβ	47.8	33.1	19.1	

**Table 3:** Physical properties of pyrolysis oil under different conditions.

Pyrolysis Conditions	550 °C, NP	550 °C, NP	520 °C, VP	550 °C, VP	650 °C, NP
S/(wt%)	0.6	0.58	0.8	1.26	1.35
H/C	1.6	1.60	1.5	1.36	1.42
Density/(kg.m^−3^)	900	900	950	987	943
Flash point/°C	20	20	28	30	<30
Heat value/(MJ/kg)	43.27	43.27	43.7	41.0	41.6
Ref.	[94]	[95]	[20]	[58]	[96]

NP: normal pressure. VP: vacuum pressure.

**Table 4:** Contents of pyrolysis oil fractions at different temperatures.

Reactors	Temperature (°C)	Fraction Content (vol%)			Ref.
		Light (<200 °C)	Medium (200–350 °C)	Heavy (>350 °C)	
FBR	475	50	45	5	[101] ^a^
FBR	500	30	55	15	[68]
FBR	550	60	35	5	[96] ^b^
MBR	600	45	35	20	[102]

FBR: fixed bed reactor; MBR: moving bed reactor. ^a^ Motorcycle tire; ^b^ car tire; n.r. not reported.

**Table 5:** Parameters of pyrolysis carbon black at different pyrolysis temperatures.

Temperature (°C)	Carbon (wt%)	Ash (wt%)	S (wt%)	Specific Surface Area (m^2^/g)	Ref.
500	82.18	14.6	3.6	43.1	[129]
550	77.22	14.58	2.41	89.1	[58]
550	88.0	13.2	2.5	65.7	[130]
550	86.3	12.5	2.8	64	[94]
600	86.6	7.10	2.10	116.3	[36]
650	82.60	14.80	2.30	63.5	[96]
700	83.0	14.8	2.7	83	[131]

## Data Availability

The data presented in this study are available on request from the corresponding author.

## Abbreviations

WT	waste tire
NR	natural rubber
SR	synthetic rubber
SBR	styrene butadiene rubber
BR	butyl rubber
MS	mass spectrometric
MRI	magnetic resonance imaging
TG-FTIR	thermogravimetric analyzer coupled with Fourier-transform infrared spectrometry
RMD	reactive molecular dynamics
GC-MS	gas chromatography-mass spectrometry
TG-MS	thermogravimetric–mass spectrometric
TGA	thermogravimetric analysis
TG	thermogravimetric
CO_x_	carbon oxides
N_2_	nitrogen
PAH	polycyclic aromatic hydrocarbons
FCC	fluid catalytic cracking
Ca(OH)_2_	calcium hydroxide
Na_2_CO_3_	sodium bicarbonate
USY	ultra-stable Y zeolite
Ru	ruthenium
LFF	light fuel fraction
NP	normal pressure
VP	vacuum pressure
HHV	high heat value
Mo	molybdenum
NiMo	nickel molybdenum
AL_2_O_3_	alumina oxide
FBR	fixed-bed reactor
FLBR	fluidized bed reactor
MBR	moving bed reactor
n.r.	not reported
H_2_	hydrogen
CO	carbon monoxide
CO_2_	carbon dioxide
CH_4_	methane
C_2_H_6_	ethane
C_2_H_4_	ethylene
C_3_H_6_	propylene
C_3_H_8_	propane
C_2_H_2_	acetylene
C_4_H_10_	butane
C_4_H_6_	1,3-butadiene
C_5_H_12_	pentane
C_6_H_6_	benzene
C_7_H_8_	toluene
C_8_H_10_	xylene
N_2_	nitrogen
H_2_S	hydrogen sulfide
ADSD	atomization dispersion and high temperature sputtering drying
